# Determination Method of Bridge Rotation Angle Response Using MEMS IMU

**DOI:** 10.3390/s16111882

**Published:** 2016-11-09

**Authors:** Hidehiko Sekiya, Takeshi Kinomoto, Chitoshi Miki

**Affiliations:** 1 Advanced Research Laboratories, Tokyo City University, 8-15-1 Todoroki, Setagaya 158-0082, Japan; 2 Maintenance and Transportation Division, Metropolitan Expressway Co., Ltd., 1-4-1 Kasumigaseki, Chiyoda-ku 100-8930, Japan; t.kinomoto86@shutoko.jp; 3 Tokyo City University, 1-28-1 Tamazutsumi, Setagaya 158-8557, Japan; cmiki@tcu.ac.jp

**Keywords:** bridge health monitoring, micro-electro-mechanical systems, inertial measurement unit, rotation angle response, angular velocity, free vibration

## Abstract

To implement steel bridge maintenance, especially that related to fatigue damage, it is important to monitor bridge deformations under traffic conditions. Bridges deform and rotate differently under traffic load conditions because their structures differ in terms of length and flexibility. Such monitoring enables the identification of the cause of stress concentrations that cause fatigue damage and the proposal of appropriate countermeasures. However, although bridge deformation monitoring requires observations of bridge angle response as well as the bridge displacement response, measuring the rotation angle response of a bridge subject to traffic loads is difficult. Theoretically, the rotation angle response can be calculated by integrating the angular velocity, but for field measurements of actual in-service bridges, estimating the necessary boundary conditions would be difficult due to traffic-induced vibration. To solve the problem, this paper proposes a method for determining the rotation angle response of an in-service bridge from its angular velocity, as measured by a inertial measurement unit (IMU). To verify our proposed method, field measurements were conducted using nine micro-electrical mechanical systems (MEMS) IMUs and two contact displacement gauges. The results showed that our proposed method provided high accuracy when compared to the reference responses calculated by the contact displacement gauges.

## 1. Introduction

**Figure 1:**
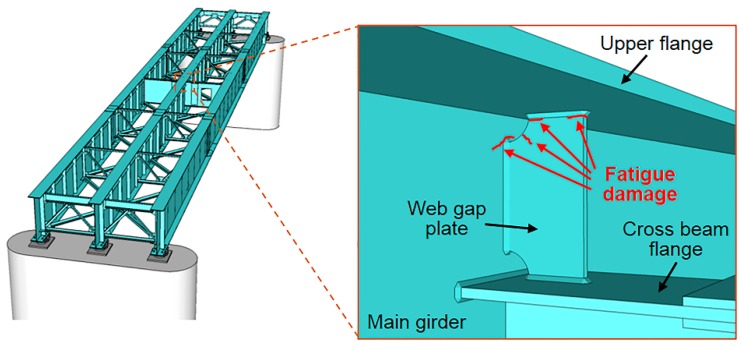
Example of fatigue damage at a steel bridge web gap plate.

**Figure 2:**
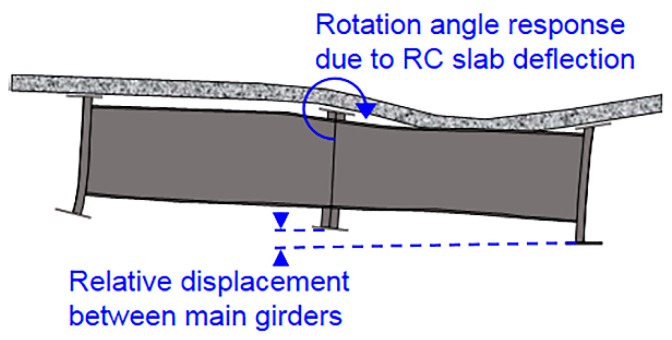
Example of the rotation angle response due to reinforced concrete (RC) slab deflection and relative displacement between main girders.

In steel bridge maintenance, countermeasures for fatigue damage are important because such damage is hard to detect and may cause sudden brittle failure. In order to conduct appropriate maintenance for fatigue damage in a steel bridge, it is important to monitor bridge deformation, which consists of the displacement response and the rotation angle response, against traffic loads because such deformation is the primary source of the stress concentrations that cause fatigue damage [1,2,3]. Figure 1 shows an example of fatigue damage at a web gap plate, which is typical fatigue damage in steel bridge. Concentrations of stress that lead to the fatigue damage at a web gap plate are thought to be caused by either the rotation angle response of the upper flange due to reinforced concrete (RC) slab deflection or the relative displacement between main girders, as shown in Figure 2. Therefore, when such fatigue damage is prevented or repaired, in the case of the former, the RC slab stiffness should be strengthened, whereas in the case of the latter, the main girder stiffness should be strengthened. Thus, gaining an understanding of bridge deformations enables the implementation of appropriate and timely maintenance and reinforcement needed to prevent fatigue damage.

Herein, due to their compact and inexpensive nature, the use of micro-electro-mechanical system (MEMS) sensors to measure responses against the external forces of traffic loading, wind force, and seismic force are proposed [4,5,6,7,8,9]. Unlike strain gauges, which have often been used to measure responses against the external forces [1], MEMS sensors can be easily attached to the painted surfaces of steel bridges using magnets. Furthermore, unlike fixed reference-based measuring tools such as linear variable differential transformers (LVDTs) [10], laser Doppler vibrometers (LDVs) [11,12], and vision-based systems [13,14,15], MEMS sensors do not require fixed reference points, which are often unavailable in large civil infrastructures [16]. Therefore, the increased use of MEMS sensors in full-scale civil infrastructures has recently been anticipated.

In order to monitor bridge deformations against actual traffic loads, bridge displacement measurements are essential, and many studies have explored methods for conducting such measurements using accelerometers [17,18,19,20,21]. However, in order to accurately monitor bridge deformation against traffic loads, it is necessary not only to measure the bridge displacement response, but also to measure the bridge rotation angle response. If a bridge structure is composed of only one material, the bridge rotation angle response can be approximately extrapolated from the displacement responses at multiple points. However, in a mixed structure, such as a steel-concrete girder bridge, it is difficult to extrapolate the bridge rotation at the joints between an upper flange and an RC deck using the displacement responses measured at that location because of differences between the stiffness of steel and RC. Therefore, the rotation angle response at joints composed of different materials should be measured at a point where we want to obtain rotation angle response data.

Hou et al. [22] proposed a method that can be used to determine bridge displacement responses using inclinometers, and the results provided in that study indicate that the proposed method is highly accurate. However, it is thought that since an in-service bridge would be constantly vibrating due to vehicle traffic, and it would be difficult to accurately measure the rotation angle response using an inclinometer because the measured data would be subject to traffic acceleration-generated bridge vibration.

In another study considering gravitational acceleration measurements, it was thought that the rotation angle could be calculated from gravitational acceleration changes caused by the MEMS accelerometer tilt [23]. However, since an in-service bridge is always vibrating due to traffic loads, changes in the measured acceleration would include changes in the gravitational acceleration and acceleration generated by bridge vibration, and it would be difficult to separate the acceleration types.

In theory, a bridge rotation angle response can be calculated from the integration of its angular velocity, as measured by an inertial measurement unit (IMU). However, the integration process would require numerical integration of the initial condition, which would be difficult to achieve on an in-service bridge that is constantly vibrating due to vehicle traffic. Furthermore, since sensor noise and quantization errors, due to the analog-to-digital (A/D) conversion process, are inevitably included in the angular velocity measured by an IMU (particularly at low frequencies), and those errors can be expected to degrade the integrated results [21].

In the present study, in order to analyze bridge rotation angle responses, field measurements were first conducted on an actual in-service bridge using nine MEMS IMUs and two contact displacement gauges. Next, based on the characteristics of the bridge rotation angle response, a method was proposed by which we could calculate the bridge rotation angle response from the measured angular velocity. Finally, by comparing the rotation angle calculated from the measured angular velocity using the proposed method with the rotation angle determined from two contact displacement gauges, the effectiveness of the proposed method was verified. The results obtained using our proposed method were found to be highly accurate.

## 2. Experimental Section

### 2.1. Description of Test Bridge and Measuring Instrument Used in This Study

**Figure 3:**
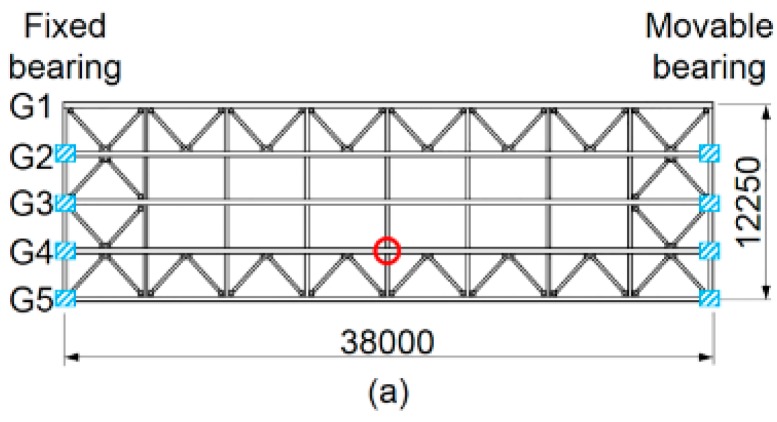

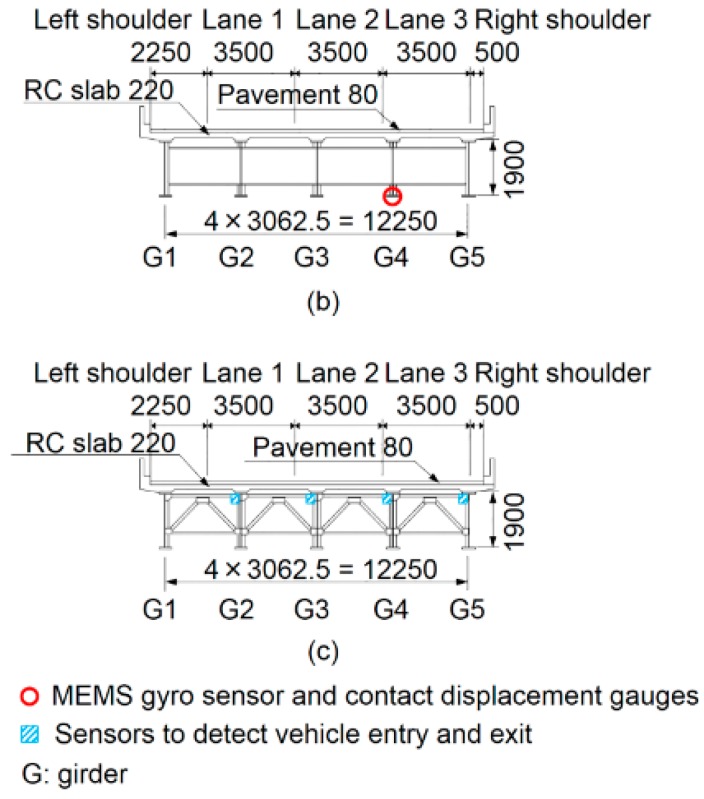
Test bridge used in our field measurements (units: mm): (**a**) top view; (**b**) front view at the longitudinal center of main girder; and (**c**) front view at the main girder edge.

Field measurements of an actual in-service bridge were performed in order to analyze the bridge rotation angle response characteristics and propose a method to determine rotation angle from measured angular velocity. The test bridge, which is a mixed steel-concrete structure equipped with three traffic lanes, is a 38-m-long single-span bridge (see Figure 3) equipped with five main 1.9-m-high girders (hereafter, G1 to G5) supporting RC deck plates. The total width of the structure is 14.25 m. The bridge is managed by the Metropolitan Expressway Co., Ltd. (Tokyo, Japan) and is located within the Tokyo Metropolitan Area.

**Figure 4:**
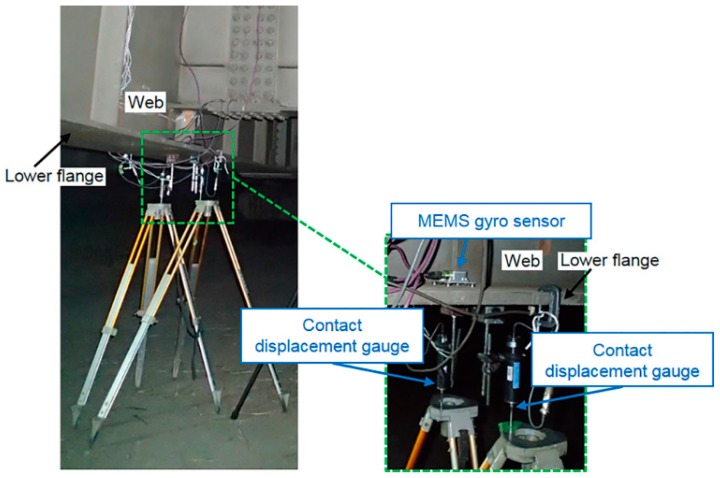
Installation of micro-electro-mechanical system (MEMS) inertial measurement unit (IMU) and two contact displacement gauges.

**Figure 5:**
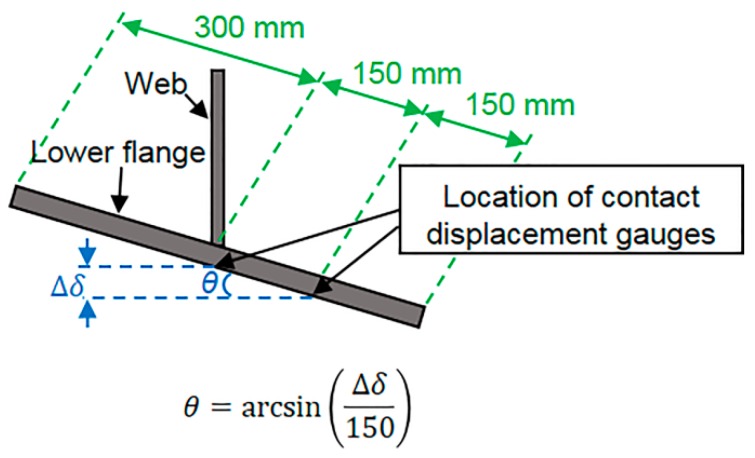
Rotation angle displacement using two contact displacement gauges.

We began by installing a MEMS IMU at the longitudinal center of the lower flange of G4 using a magnet. Two contact displacement gauges were also fixed at the same location in order to verify the accuracy of the rotation angle values obtained from the measured angular velocity. This experimental setup is shown in Figure 4. The method used to determine the bridge rotation angle values from the contact displacement gauge responses is shown in Figure 5.

**Figure 6:**
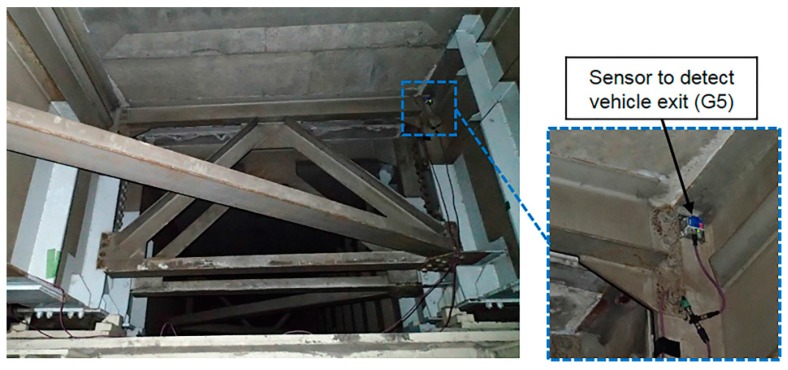
Installation of sensor to detect vehicle exit at main girder five (G5).

In order to precisely detect vehicle entry and exit times using the measured acceleration responses, eight MEMS IMUs were attached to the vertical stiffeners on both longitudinal edges of the four main girders. These IMUs also allowed us to determine the travel lanes used when vehicles passed over the bridge. The MEMS IMU experimental setup located at the vertical stiffener of the exit side of G5 is shown in Figure 6. The specifications of the MEMS IMUs and contact displacement gauges are listed in Table 1 and Table 2.

### 2.2. Analysis of Bridge Rotation Angle Response under Traffic Loads

By comparing the bridge displacement and bridge rotation angle responses under traffic load conditions in both the time and frequency domains, the bridge rotation angle response under traffic loads can be analyzed.

**Figure 7:**
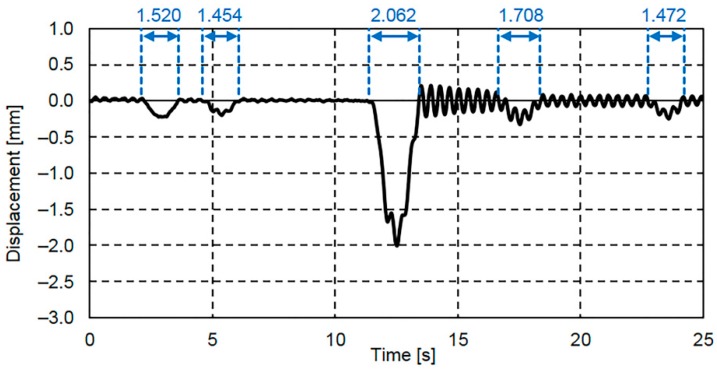
Displacement response obtained using the contact displacement gauge at G4.

The 25 s displacement response measured by the contact displacement gauges at the lower flange center of G4 is shown in Figure 7. In order to eliminate any effects other than the displacement response, Figure 7 shows the displacement response obtained after the application of a 10 Hz low-pass filter, which was selected because the main frequency range of the bridge displacement under traffic load conditions is below 10 Hz [21]. Figure 7 shows some deflections caused by the weight of vehicles travelling on the bridge. Vehicle passage times, which depend on the vehicle speed and the bridge span length, ranged from 1.454 to 2.062 s. Based on those times, a deflection frequency of approximately 0.5 to 0.7 Hz could be calculated.

**Figure 8:**
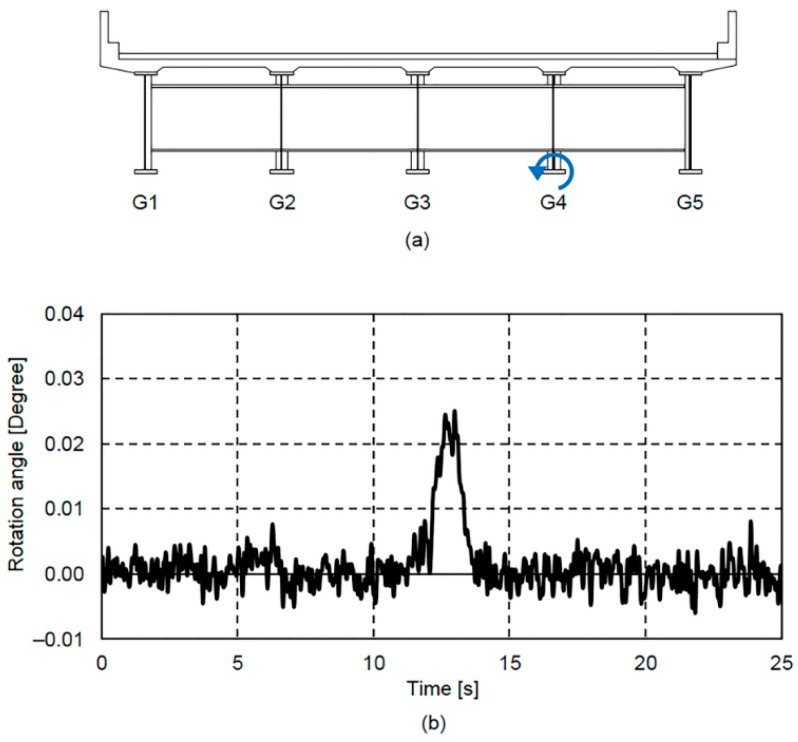
Normal direction of rotation angle response and rotation angle response obtained using two contact displacement gauges at G4: (**a**) normal direction of rotation angle response; and (**b**) rotation angle response.

The bridge rotation angle response for 25 s calculated from the displacement responses measured by the two contact displacement gauges at G4 is shown in Figure 8. One of the displacement responses is shown in Figure 7. Figure 8a shows the normal rotation angle response direction.

The displacement response in Figure 7 and the rotation angle response in Figure 8b showed similar shapes, particularly around the 12 s mark. In addition, it can be seen that when the bridge displacement response in Figure 7 is vibrating about the zero-axis, the bridge rotation angle in Figure 8b is also rotating about the zero-axis. In other words, it is thought that when no vehicle is traveling on the bridge, the bridge rotation angle is rotating about the zero-axis.

**Figure 9:**
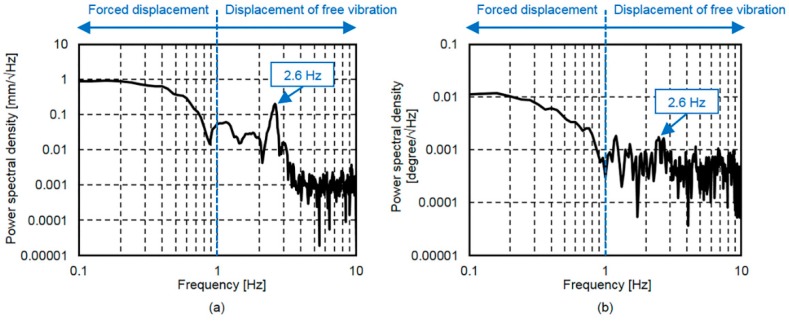
Detailed bridge response spectrum at G4: (**a**) displacement response spectrum; and (**b**) rotation angle response spectrum.

Details of the displacement response spectrum shown in Figure 7 and the rotation angle response spectrum shown in Figure 8b are shown together in Figure 9.

The displacement response spectrum in Figure 9a and the rotation angle response spectrum in Figure 9b also show similar shapes, particularly at the frequencies below 1.0 Hz, which is due to forced displacement. As can be seen in Figure 9a, the displacement response spectrum shows a natural vibration frequency at 2.6 Hz. In addition, while the rotation angle response spectrum peak shown in Figure 9b is not as well-defined, it also shows a 2.6 Hz natural vibration frequency. In the frequency range of 4 Hz to 10 Hz, neither spectrum shows other well-defined higher order mode peaks that depend on vibration. Therefore, it can be said that, based on our comparison between the displacement and rotation angle response spectra, as with the displacement response [21], the rotation angle response is largely classified into forced displacement and free vibration displacement responses.

## 3. Calculation Method for Determining Bridge Rotation Angle from Measured Angular Velocity

As described above, the rotation angle response is largely classified into forced displacement and free vibration displacement responses. In addition, when no vehicle is travelling on the bridge, the bridge rotation angle rotates about the zero-axis. Using these characteristics, the bridge rotation angle response is determined from the measured angular velocity. The basic concept of our proposed method will now be described. First, precise vehicle entry and exit times are detected using acceleration responses recorded by MEMS IMUs at the vertical stiffeners on both longitudinal edges of the main girders. Second, by assuming that the bridge is rotating at its free vibration frequency before and after the vehicle travels over the bridge, the initial and terminal conditions of the rotation angle response can be estimated. Finally, the angular velocity of the bridge during vehicular passage is integrated into the rotation angle response using the initial and terminal estimates produced above.

**Figure 10:**
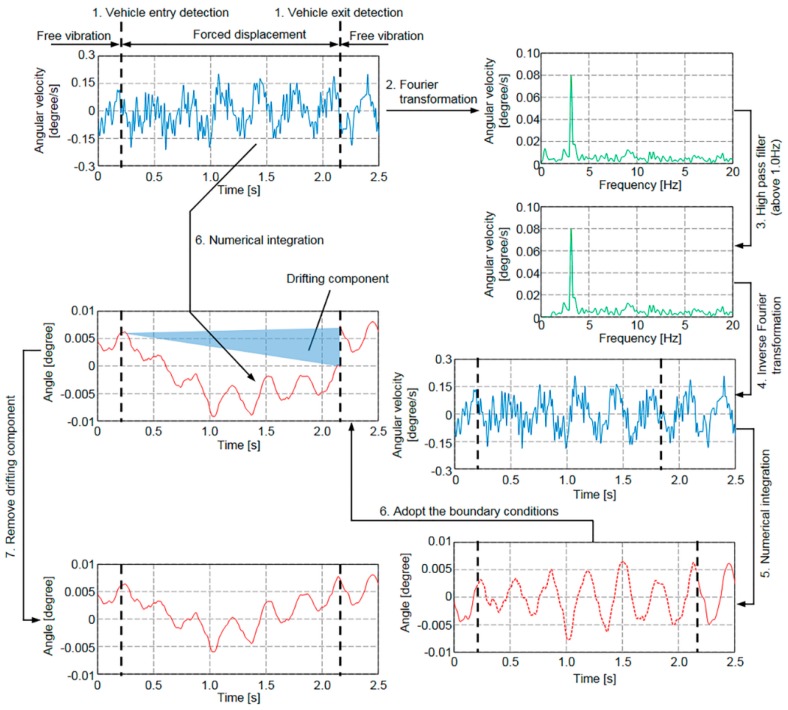
Procedure used in the proposed method to determine bridge rotation angle from measured angular velocity.

The procedure used by the proposed method to determine the bridge rotation angle response from the angular velocity measured by the IMUs (see Table 1) is shown in Figure 10. It consists of the following steps:

1. Free and forced vibration regions are separated by precisely detecting vehicle entry and exit times. When multiple vehicles are running on the bridge, the time the first vehicle enters the bridge is the vehicle entry time and the time the last vehicle exits the bridge is the vehicle exit time.
2. The angular velocity is transformed from the time domain to the frequency domain through a Fourier transform.
3. Frequencies below 1.0 Hz are removed in order to eliminate the effect of forced displacement during vehicular passage. Although the proper high-pass filter cut-off frequency depends on the bridge length and vehicle travelling speed, since the span lengths of most girder bridges in Japan are more than 30 m and the maximum allowed speed on expressways in Japan is 100 km/h, the travelling time should be above 1.2 s, and the frequency should be below 0.9 Hz [21]. In addition, since the natural frequency of this test bridge in Figure 9 is 2.6 Hz, the cut-off frequency of the high-pass filter used in this study is 1.0 Hz.
4. The angular velocity in the frequency domain, which does not have the effect of forced displacement during vehicular passage, is transformed to the time domain through an inverse Fourier transform.
5. The angular velocity under free vibration conditions obtained in Step 4, which excludes the effects of vehicular forced displacement, is numerically integrated to obtain the rotation angle, thereby producing an estimate of the initial and terminal rotation angle conditions under free vibration conditions.
6. The total angular velocity, which includes the angular velocity under both free and forced vibration conditions, is numerically integrated to obtain the total rotation angle. The lower and upper limits of the integration are the times when the same vehicle enters and exits the bridge, respectively. The initial rotation angle is defined as the rotation angle under free vibration conditions estimated in Step 5.
7. Because of measurement errors in the measured angular velocity, which are caused by sensor noise and the limitations of A/D conversion, the integrated rotation angle does not satisfy the terminal conditions at this point. To satisfy those conditions, the drift component is subtracted from the integrated rotation angle.

In the procedure described above, when the initial and terminal conditions of the rotation angle response in Step 3 cannot be accurately estimated, the integrated rotation angle response results include the estimated error of the initial and terminal conditions. Therefore, vehicle detection in Step 1 is of crucial importance. 

The measurement error, particularly the low frequency range measurement error, which includes sensor noise and quantization errors due to the A/D conversion process, corrupts the integrated results. Furthermore, since the generated angular velocity obtained over the low frequency range is small compared to the generated angular velocity over the high frequency range, low frequency range measurement data is easily affected by sensor noise. Therefore, it is important to use an IMU with low sensor noise over the low frequency range so that the angular velocity can be measured over that range with a high level of accuracy. The noise density of an IMU used in this study is 0.004 [(°/s)/√Hz]. In Figure 10, the generated angular velocity is about ±0.15 [°/s]. As a result, it is thought that the noise density is sufficiently small for the generated angular velocity of a bridge-axial-orthogonal direction. 

## 4. Results and Discussion

In order to verify the effectiveness of the method used to determine the bridge rotation angle response from the measured angular velocity, comparisons between the rotation angle response determined from the measured angular velocity and those determined by the two contact displacement gauges were conducted.

### 4.1. Test Procedure

The test bridge and instrument installation positions are the same as those shown in Figure 3. The field measurements were performed without traffic control using two contact displacement gauges and nine MEMS IMUs, which included a MEMS IMU for measuring angular velocity at the longitudinal center of the lower flange of G4 and eight MEMS IMUs for detecting vehicle entry and exit times at the vertical stiffeners on both longitudinal edges of the four main girders. The test procedures are as follows:

1. Vehicle entry and exit times are detected by eight MEMS IMUs at the vertical stiffeners on both longitudinal edges of the main girders.
2. The angle rotation response at the longitudinal center of the lower flange of G4 is determined from the measured angular velocity at the longitudinal center of that lower flange by using the detected vehicle entry and exit times and the proposed method.
3. Accuracy verification is conducted by comparing the rotation angle response determined from the measured angular velocity and those determined by the two contact displacement gauges.

### 4.2. Vehicle Detection Results Using Eight MEMS IMUs

**Figure 11:**
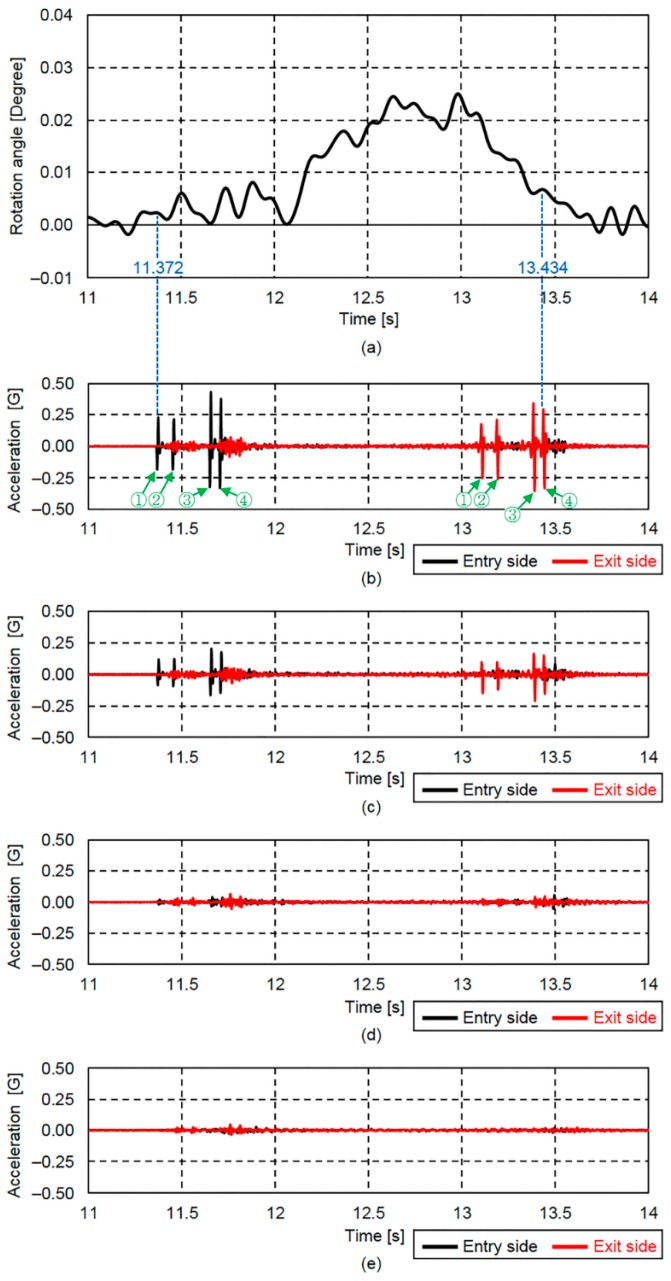
Vehicle entry and exit detection times based on acceleration records at the vertical stiffener both longitudinal edges of the main girder: (**a**) rotation angle response obtained using the two contact displacement gauges; (**b**) acceleration records at G2; (**c**) acceleration records at G3; (**d**) acceleration records at G4; and (**e**) acceleration records at G5.

In order to apply the proposed method, precise vehicle entry and exit times must be measured. In this study, the eight above-mentioned MEMS IMUs were used for this purpose. In addition, the vehicle travel lane was estimated using each of the acceleration responses recorded at G2 to G5 by the eight MEMS IMUs. The determined rotation angle response using two contact displacement gauges, and each acceleration response measured at G2 to G5 are shown in Figure 11.

The acceleration record in Figure 11b revealed that the passage of a four-axle vehicle with an entry time of 11.372 s and an exit time of 13.434 s. It can be seen that the rotation angle response in Figure 11a starts to increase at the vehicle entry time (11.372 s). Furthermore, since the G2 acceleration response for the entry and exit sides showed the highest value, and the acceleration G3 response value for the entry and exit sides showed the second highest value, the four-axle vehicle could be presumed to be traveling on Lane 1 (see Figure 3c).

### 4.3. Verification of the Rotation Angle Response Determined from Measured Angular Velocity

By comparing the rotation angle response determined from the measured angular velocity with that determined by the two contact displacement gauges, the accuracy of the response determined from the measured angular velocity could be verified. Then, by using an entry time (11.372 s) and an exit time (13.434 s) as the limits of the numerical integration, the rotation angle response was determined from measured angular velocity.

**Figure 12:**
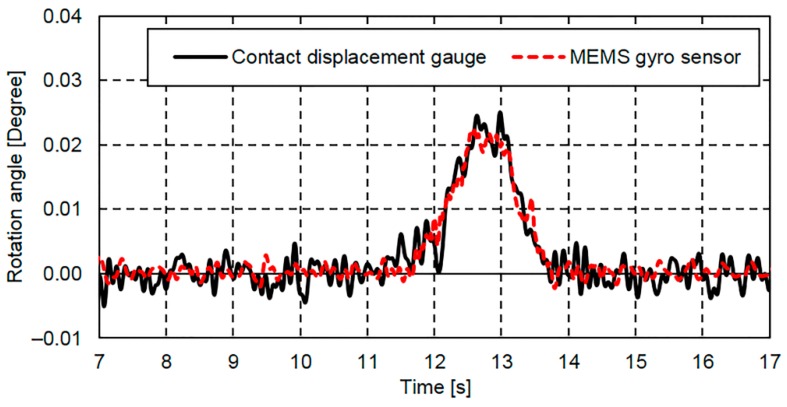
Rotation angle response obtained via MEMS IMU at G4 using the proposed method.

The rotation angle response determined from the MEMS IMU and that determined by the two contact displacement gauges also placed at G4 are shown in Figure 12, where it can been seen that both responses start to increase at the vehicle entry time (11.372 s) and both lines show similar shapes. Based on these results, the proposed method was found to be highly accurate when compared with the reference rotation angle response produced using the two contact displacement gauges.

It can be seen that the rotation angle response determined by the two contact displacement gauges contains more high-frequency components than that determined from the MEMS IMU. It is thought that this is caused by electric noise included in the displacement response measured by the contact displacement gauges.

## 5. Conclusions

In the present study, by conducting field measurements of an actual in-service bridge, the characteristics of the bridge rotation angle response were analyzed first. Next, based on the bridge rotation angle response characteristics, a method for determining the bridge rotation angle response from the measured angular velocity was proposed. Then, in order to detect the precise vehicle entry and exit times and assess the vehicle travel lane, a vehicle detection system using eight MEMS IMUs was constructed. Finally, by determining the bridge rotation angle response from the measured angular velocity using the proposed method, the effectiveness of the method was verified. The conclusions of the present study are as follows:

- It was found that, as with the displacement response, the rotation angle response is largely classified into the response due to forced displacement, which consists of frequencies below 1.0 Hz, and the free vibration response. This result was achieved as a result of comparisons between the bridge displacement response spectrum and the bridge rotation angle response spectrum.
- Based on the bridge rotation angle response characteristics, which are largely classified into the rotation angle responses due to forced displacement and free vibration displacement responses, a method for determining the bridge rotation angle response from the measured angular velocity was proposed.
- Vehicle entry and exit times and the vehicle travelling lane could be estimated using acceleration responses measured by eight MEMS IMUs. This system worked by comparing the maximum acceleration response values measured at the longitudinal edges of each main girder.
- The determined rotation angle responses using IMU and the proposed method were found to be in good agreement with the reference rotation angle responses calculated by the two contact displacement gauges. This outcome was obtained by examining the results of field measurements taken from an in-service bridge using nine IMUs and two contact displacement gauges.

In this study, by using vehicle passage time as the limits of the numerical integration, the rotation angle response was determined from the measured angular velocity. Further studies are required to verify the effectiveness of the proposed method for multiple vehicle travelling conditions. Furthermore, this study focuses solely on the bridge-axial-orthogonal direction rotation angle response. Further studies will be required to verify the effectiveness of the proposed method for the bridge-axial direction rotation angle response and the bridge rotation angle response at multiple points.

## Figures and Tables

**Table 1:** Specifications of MEMS IMUs.

IMU	Measurement Range	Frequency Bandwidth	Sampling Frequency	Resolution	Noise Density
M-G550-PC (SEIKO EPSON Co., Ltd. (Suwa, Japan))	±300 [°/s]	133 (−3 dB) [Hz]	100 [Hz]	0.0125 [(°/s)/LSB]	0.004 [(°/s)/√Hz]
M-G550-PC (SEIKO EPSON Co., Ltd. (Suwa, Japan))	±29.4 [m/s^2^]	148 (−3dB) [Hz]	500 [Hz]	1226 [μm/s^2^]	981 [μm/(s^2^√Hz)]

**Table 2:** Contact displacement gauges.

Contact Displacement Gauge	Capacity [mm]	Sensitivity [×10^–6^ Strain/mm]	Nonlinearity [mm]	Sampling Frequency [Hz]
CDP-50 (Tokyo Sokki Kenkyujo Co., Ltd. (Tokyo, Japan))	0–50	200	0.1% RO	100

## Abbreviations

The following abbreviations are used in this manuscript:

IMU	inertial measurement unit
MEMS	micro-electro-mechanical systems
LDVT	linear variable differential transformer
LDV	laser Doppler vibrometer
RC	reinforced concrete

## References

[1] Fisher J.W. (1984). Fatigue and Fracture in Steel Bridges.

[2] Fisher J.W., Fisher T.A., Kostem C.N. (1979). Displacement induced fatigue cracks. Eng. Struct..

[3] Gan W., Hu W., Liu F., Tang J., Li S., Yang Y. (2016). Bridge Continuous Deformation Measurement Technology Based on Fiber Optic Gyro. Photonic Sens..

[4] Pakzad S.N., Fenves G.L. (2009). Statistical analysis of vibration modes of a suspension bridge using spatially dense wireless sensor network. J. Struct. Eng..

[5] Rice J.A., Mechitov K.A., Sim S.H., Spencer B.F., Agha G.A. (2011). Enabling framework for structural health monitoring using smart sensors. Struct. Health Monit..

[6] Lynch J.P., Wang Y., Loh K.J., Yi J.-H., Yun C.-B. (2006). Performance monitoring of the Geumdang Bridge using a dense network of high-resolution wireless sensors. Smart Mater. Struct..

[7] Shinozuka M., Papakonstantinou K.G., Torbol M., Kim S. (2015). Real-time remote monitoring: The DuraMote platform and experiments towards future, advanced, large-scale SCADA systems. Struct. Infrastruct. Eng..

[8] Ha D.W., Park H.S., Choi S.W., Kim Y. (2013). A Wireless MEMS-based inclinometer sensor node for structural health monitoring. Sensors.

[9] Nagayama T., Moinzadeh P., Mechitov K., Ushita M., Makihata N., Ieri M., Agha G., Spencer B.F., Fujino Y., Seo J.W. (2010). Reliable multi-hop communication for structural health monitoring. Smart Struct. Syst..

[10] Gindy M., Vaccaro R., Nassif N. (2008). A state-space approach for deriving bridge displacement from acceleration. Comput. Aided Civ. Inf..

[11] Nassif H.H., Gindy M., Davis J. (2005). Comparison of laser Doppler vibrometer with contact sensors for monitoring bridge deflection and vibration. NDT E Int..

[12] Miyashita T., Fujino Y. (2007). Development of three-dimensional measurement system using laser Doppler vibrometers. J. Jpn. Soc. Civ. Eng. A.

[13] Yoneyama S., Kitagawa A., Iwata S., Tani K., Kikuta H. (2007). Bridge deflection measurement using digital image correlation. Exp. Tech..

[14] Lee J.J., Shinozuka M. (2006). A vision-based system for remote sensing of bridge displacement. NDT E Int..

[15] Yoneyama S., Ueda H. (2012). Bridge deflection measurement using digital image correlation with camera movement correction. Mater. Trans..

[16] Cho S., Sim S.-H., Park J.-W., Lee J. (2014). Extension of indirect displacement estimation method using acceleration and strain to various types of beam structures. Smart Struct. Syst..

[17] Park K.-T., Kim S.-H., Park H.-S., Lee K.-W. (2005). The determination of bridge displacement using measured acceleration. Eng. Struct..

[18] Gindy M., Nassif H.H., Velde J. (2007). Bridge displacement estimates from measured acceleration records. J. Transp. Res. Board.

[19] Park J.-W., Sim S.-H., Jung H.-J. (2014). Wireless displacement sensing system for bridges using multi-sensor fusion. Smart Mater. Struct..

[20] Park J.-W., Sim S.-H., Jung H.-J., Spencer B.F. (2013). Development of a wireless displacement measurement system using acceleration responses. Sensors.

[21] Sekiya H., Kimura K., Miki C. (2016). Technique for determining bridge displacement response using MEMS accelerometers. Sensors.

[22] Hou X., Yang X., Huang Q. (2005). Using inclinometers to measure bridge deflection. J. Bridge Eng..

[23] Sugisaki K., Abe M., Koshimizu S. (2012). Inclination and vibration measurement by inertial sensing for structural health monitoring. J. Jpn. Soc. Civ. Eng. A1.

